# Building an ECMO/ECPR Pathway—Operational Metrics and Patient Outcomes in One Year

**DOI:** 10.3390/jcm15020912

**Published:** 2026-01-22

**Authors:** Edgars Prozorovskis, Katrina Loceniece, Davis Polins, Eva Strike

**Affiliations:** 1 Department of Cardiovascular Anesthesia and Intensive Care, Pauls Stradins Clinical University Hospital, LV-1002 Riga, Latvia; davis.polins@stradini.lv (D.P.); eva.strike@stradini.lv (E.S.); 2 Faculty of Medicine, Riga Stradins University, LV-1007 Riga, Latvia; kloceniece@yahoo.com; 3 Department of Anesthesiology, Riga Stradins University, LV-1007 Riga, Latvia

**Keywords:** ECMO, ECPR, operational metrics, patient outcomes, in-hospital cardiac arrest, post-insertion complications, comorbidities, extracorporeal membrane oxygenation, extracorporeal cardiopulmonary resuscitation

## Abstract

**Background/Objectives:** Pauls Stradins Clinical University Hospital in Riga, Latvia, introduced an ECMO program in 2008. Since the program’s start, countless patients have had their lives saved by this necessary technology. Our goal was to review the ECMO program results and gain insight into the organization’s operations. We wanted not only to assess the program’s efficiency in terms of time, but also to visualize patient outcomes at least a month after decannulation from ECMO and discharge from the hospital. **Methods:** A retrospective observational study was performed using hospital patient data files from October 2024 to October 2025. The selected patient group was those who had suffered an in-hospital cardiac arrest and successfully had ECMO inserted; this criterion fit fifteen patients. Data were collected on multiple factors, including from collapse to flow time, the number of days spent in the ICU, and post-ECMO complications. Afterwards, the data were analyzed to understand the program’s and patients’ outcomes. **Results:** Of the fifteen patients analyzed, seven did not survive to hospital discharge. The statistically significant quantitative results were the first lactate levels after ECMO cannulation and the first troponin levels after cardiac arrest. In terms of qualitative results, CHF, survival to ECMO decannulation, cannulation failure, and survival to ICU discharge were statistically significant. **Conclusions:** The ECMO program at Pauls Stradins Clinical University Hospital provides patients with a necessary technology after an intra-hospital cardiac arrest. This study highlights data about these patients and their outcomes, as well as areas for improvement within the hospital’s ECMO/ECPR program.

## 1. Introduction

Since the invention of extracorporeal membrane oxygenation in 1974 [1], patients in critical condition whose hearts and/or lungs are not working have been given a chance to survive. ECMO became more widely used during the H1N1 influenza epidemic in the 2000s, mainly due to the prevalence of ARDS [2]. ECMO uses arterial and venous cannulation to provide oxygenation and pump blood through the circulatory system. This process delivers essential oxygen and nutrients to cells, organs, and tissues. The use of this technology for a patient can last from hours to months. The duration depends on many factors, including bleeding, infections, lab results, and the prognosis for survival. There are two types of ECMO: venovenous and venoarterial (which is further divided into: central, peripheral, and veno-arterial-venous) [3]. Venoarterial ECMO supports both cardiac and pulmonary function. Venovenous ECMO supports only pulmonary function. Venoarterial ECMO returns oxygenated blood to the aorta, bypassing both the heart and the lungs. Venovenous ECMO returns blood to the right atrium, so the heart can still pump on its own [3]. This study analyzes the use of venoarterial ECMO during IHCA in our program.

In Latvia, two hospitals offer ECMO technologies: Pauls Stradins Clinical University Hospital (PSKUS) and Riga East Clinical University Hospital. PSKUS is considered the central location for ECMO placement and advancements in the country and the city. The first use of ECMO in Latvia was in 2008 at PSKUS. This patient had repeated cardiac surgery, with aortic and mitral valve prostheses installed. There was a septal wall defect, so traditional cardiac bypass was not an option. Therefore, a central ECMO was placed. Until 2021, the ECMO placement was conducted mainly in the cardiac surgery department, using its resources, with up to 30 patients per year. According to international health guidelines, an ECMO center should serve at least 10 patients per million inhabitants per year to be considered highly successful [4,5]. In November 2021, it was decided to expand the ECMO program to treat other patients—not just those undergoing or recovering from cardiac surgery, but also, for example, patients on CRRT. This push for expansion has allowed ECMO to be placed not only in operating rooms but also in hybrid operating rooms, the ICU, the ER, and within departments. Although ECMO development in Latvia is ongoing, its most significant barrier remains the high cost.

Cardiac arrest does not have a favorable prognosis. However, the mortality rate for intra-hospital cardiac arrest is decreasing [6]. IHCA is defined as the delivery of compressions and/or defibrillation in a hospital. It is compared to inpatient beds, according to the Utstein resuscitation registry [7]. In other countries, like the United States, there is an average of 10.6 IHCAs per 1000 patients [8]. According to current European data, survival rates for IHCA range from 15% to 34%. For out-of-hospital cardiac arrest (OHCA), the chance of survival is about 8% [9]. Time is critical for neurological outcomes after cardiac arrest. After 15–20 min, the prognosis is poor [10]. In hospitals, resuscitation begins faster than outside a healthcare facility. Hospitals have specialized equipment, trained personnel, standard algorithms to follow, and a more controlled environment overall.

## 2. Materials and Methods

### 2.1. Study Type

A retrospective observational study was planned (i.e., no control group for comparison). This research type was suitable for our goals, as it would allow us to reflect on and analyze past events. Since intra-hospital cardiac arrest (IHCA) is unpredictable and not every patient meets ECMO criteria, it was decided that this study design would be best for this article. Whilst reviewing the patient files, it was concluded that analyzing ECMO patients with IHCA would be more beneficial than analyzing OHCA cases. This action yielded more documentation and more precise results, as documentation is more rigorous within the hospital.

### 2.2. Inclusion and Exclusion Criteria

Criteria for inclusion in the study were

(1) adults over the age of 18 years old,
(2) patients receiving treatment at PSKUS in Riga, Latvia,
(3) patients who suffered an intra-hospital cardiac arrest,
(4) patients who received active resuscitation efforts,
(5) patients who had venoarterial ECMO successfully placed and were attached to the system for at least an hour,
(6) survival expected for more than six months.

Exclusion criteria were

(1) out-of-hospital cardiac arrest (due to gaps in documentation and information required for the study),
(2) unwitnessed cardiac arrest,
(3) circulatory death within an hour of placement on ECMO,
(4) placement of solely venovenous ECMO,
(5) cardiac arrest not due to pulmonary or cardiac causes (such as due to sepsis or trauma, for example),
(6) patients who consciously denied their data being used for research purposes,
(7) traumatic cardiac arrest patients (such as those brought in after an MVA),
(8) cardiac arrest patients succeeding unsurvivable conditions (such as massive intracranial hemorrhage or terminal malignancies),
(9) special patient groups such as pregnant patients or children,
(10) patients with substantial missing data in their patient files, which were required for the study.

### 2.3. Cannulation at PSKUS and Latvian Guidelines

Data were obtained from Pauls Stradins Clinical University Hospital in Riga, Latvia. Although ECMO was first introduced at this hospital in 2008, a specialized team was formed in 2024. When a CA occurs at PSKUS, the on-call ECMO team is notified and has up to one hour to respond. The foremost ECMO specialists are certified anesthesiologists/reanimatologists with extensive training in ECMO placement. Additional team members may include cardiothoracic surgeons, anesthesiologists, cardiologists, nurses, and/or perfusionists. ECMO is usually chosen for CA cases that are suspected to be cardiac or pulmonary in origin; examples include acute coronary syndrome or pulmonary embolism. Hospital protocol is followed for resuscitation measures, such as administering epinephrine doses, notifying the appropriate staff, and more, prior to ECMO placement. At this hospital, ECMO technology can be placed in various locations, including operating rooms, the emergency department, catheterization rooms (hybrid operating rooms), and intensive care units.

The ECMO team at PSKUS is activated within five minutes of cardiac arrest if it is deemed necessary. However, at our center, there is no specific number of defibrillations or minutes within initiation of CPR to call the ECMO team. This is to prevent the use of this technology too late for a favorable patient outcome. The one-hour time frame used at PSKUS is the time frame until ECMO flow. The decision to use this technology is always made within minutes of cardiac arrest. The implantation decision is made by an on-site doctor, but the final decision is made by the ECMO specialist. In terms of ECMO indication criteria in Latvia, the Latvian State Agency of Medicine has defined criteria for ECMO placement, including and excluding patients. The absolute contraindications for venoarterial and venovenous ECMO are

(1) irreversible multiorgan damage or neurological illness,
(2) catastrophic heart/liver/lung pathology,
(3) 60 full minutes of resuscitation efforts (CPR) without ROSC,
(4) contraindications for blood transfusion or anticoagulant therapy,
(5) intracranial hemorrhage or recent neurosurgery within the last ten days,
(6) severe chronic pulmonary hypertension with pulmonary artery pressure over 50 mmHg,
(7) terminal oncology patients with low chances of survival,
(8) FiO_2_ < 100 with the patient on a ventilator for over ten days, with the maximum MPV parameters in place,
(9) immunosuppressed patients with an absolute neutrophil count under 400/mm^3^ [11].

PSKUS hospital guidelines for ECMO initiation are

(1) Age under 70 years (relative),
(2) “no flow” interval under five minutes,
(3) from cardiac arrest to ECMO flow < 60 min,
(4) EtCO_2_ > 10 mmHg during CPR,
(5) no known aortic valve insufficiency,
(6) clinical reason for cardiac arrest, with opportunity for reversal of the cause.

The Latvian guidelines are based on the Extracorporeal Life Support Organization’s guidelines for ECMO and resuscitation measures. Similar examples to the Latvian guidelines include preexisting conditions incompatible with life (such as malignancies) or conditions incompatible with life (such as aortic dissection) [12]. According to Latvian guidelines, the indication criteria for ECMO are coronary vascular pathologies as well as arterial hypoxemia and hypercapnia. The common reasons for ECMO placement, which fall under the coronary vascular pathologies listed in the Latvian guidelines, are

(1) refractory cardiogenic shock,
(2) heart failure after cardiotomy,
(3) post-cardiac transplant or post-dual cardiac and lung transplant heart failure,
(4) overdose from cardiotoxic medication,
(5) myocarditis,
(6) sepsis with acute heart failure,
(7) pulmonary arterial embolism,
(8) acute refractory anaphylactic shock,
(9) cardiac arrest with cardiopulmonary resuscitation,
(10) branching for heart transplant as a mechanical support system [11].

Thus, in this study, all patients who had ECMO placed were in accordance with the indication criteria, having a cardiac arrest with subsequent cardiopulmonary resuscitation. The patients also had no absolute contraindications according to local guidelines. Pauls Stradins Clinical University Hospital uses these Latvian guidelines as the basis for ECMO placement.

It should also be noted that ECMO implantation at this hospital is performed percutaneously under ultrasound guidance. If possible and needed, fluoroscopy can also be used. If this is insufficient, echocardiography is used to assist with cannula positioning and depth analysis. In our center, the average cannulation time is 20 min.

### 2.4. Data Collection

The data were requested from the PSKUS patient data file department. All patients had signed consent forms upon arrival at the hospital, stating that their data could be used for research, as the hospital is a clinical university hospital. For those in an unstable condition who were unable to sign the consent form themselves, the forms were accepted on their behalf under the principle of implied consent.

After receiving the patient files, the research team met to discuss which categories they would like to analyze in the data and input into an Excel sheet on the computer. All the data were collected solely from physical paper copies of the patient files, where the pertinent information needed was found. For example, this could include ECGs, lab results, blood gas analysis printouts, and more. In terms of the Excel sheet, basic data such as first and last name, personal code, and patient history number were collected to help track each patient. Then, key data for each patient, including their available health history, were entered into the data table. These categories included age, gender, and BMI. Furthermore, these categories were divided into Boolean variables (yes/no) for the following health conditions: diabetes mellitus, COPD, malignancies, previous myocardial infarction, dyslipidemia, immunosuppression, previous stroke, chronic kidney disease, chronic heart failure, hypertension, and smoking status. Then, the primary diagnoses were documented for this hospital visit, along with any complications. Furthermore, this was classified into acute diagnoses, planned surgeries/procedures, and open-heart surgery. Continuing, events during CPR and some data on resuscitation efforts were analyzed, including initial cardiac rhythm before cardiac arrest, cardiac rhythm before ECMO, and epinephrine dose during resuscitation. In terms of cardiac rhythms, any of the rhythms noted before the start of ECMO were included in the data. The options included asystole, ventricular tachycardia, ventricular fibrillation, pulseless electrical activity, or mixed (meaning multiple rhythms were noted, such as ventricular fibrillation and asystole).

Moving on to the ECMO portion, data were collected on collapse time (from CA to ECMO start), cannulation site, and team composition. Subsequently, quantitative data on clinical biomarkers (lactate, pH, and troponin) were collected post-cardiac arrest. Furthermore, the lactate levels and pH after ECMO were recorded in the data table. Then, lactate, pH, and troponin levels 24 h after CA were entered. At PSKUS, lactate levels are measured in mmol/L (with a reference interval of 0.5 to 2.2 mmol/L) and high-sensitivity troponin I (hs-TnI) in ng/L (with a reference range of 0–38 ng/L). Additionally, the pH reference range is 7.35 to 7.45. To analyze survival in our center’s ECMO patients, data were dichotomized (yes/no): survival to ECMO decannulation, survival to ICU discharge, survival to hospital discharge, 30-day survival after ECMO initiation, and three-month survival after ECMO implantation. Other data collected were whether there was cannulation failure, number of days spent in the ICU, cerebral performance score (CPC) at discharge (one to five), bleeding according to the BARC classification (type 2–5), whether because of ECMO there was stroke, limb ischemia, infection, acute kidney injury (AKI), or CRRT was needed. For statistical analysis, some data were simplified, such as original diagnoses being categorized into groups, and some data were excluded, such as team composition, due to the complicated analysis of this parameter.

### 2.5. Statistical Analysis

A thorough statistical analysis of the data was conducted using IBM SPSS, version 29.0. The main aim of interpreting the results was to obtain the *p*-value; if *p* < 0.05, the data are significant. For the quantitative data, the Mann–Whitney U test was performed since a comparison of two groups, patients who survived and those who did not, was needed. In addition, the data were unpaired, and the sample size was less than 30; thus, this test was chosen. For the qualitative data, Fisher’s exact test was used because there are two unpaired categories (survivors and non-survivors) and the sample size is small, which would make the chi-squared test less reliable. Within this data set, the data were divided into patients who survived to hospital discharge and those who did not.

## 3. Results

The results were entered into an Excel sheet, where multiple tables were created, which will be displayed later. Numerical data were analyzed and are presented in Table 1. Qualitative data were analyzed and placed into Table 2 and Table 3. Firstly, data will be analyzed (15 patients) and then differentiated between patients who survived (8 patients) and those who did not (7 patients). Patients who did not survive, in this study, were defined as those who had spontaneous cardiac arrest after being placed on ECMO, and no resuscitation efforts were made.

### 3.1. Information About Patients’ Overall Health

The average age of ECMO patients in this study was 65.0 years. As for BMI, the average was 27.8 kg/m^2^. Of the 15 patients, 3 were female, and 12 were male. More specific health conditions can be seen in Table 2. Regarding patients’ diagnoses and reasons for hospitalization, 8 were acute PCA patients (e.g., myocardial infarction), 4 were elective PCA patients (e.g., ICD change to CRT-D), and 3 were post-cardiac surgery patients (e.g., aortic valve replacement).

### 3.2. Cardiac Arrest Data

Analysis of resuscitation events was also conducted. Prior to CA, 14 patients had sinus rhythm, and 1 had atrial fibrillation. The average dose of epinephrine given during resuscitation measures was 4 mg. Low-flow time, time from collapse (cardiac arrest) to ECMO flow start, on average, was 44 min. For cardiac rhythms before ECMO, the results can be seen in Table 3. It is important to note that mixed rhythm occurs when not solely one cardiac rhythm is noted. The first lactate level post-arrest mean was 7.45 mmol/L. The first pH post-arrest mean was 7.13. The first post-arrest troponin average was 6554.94 ng/L.

### 3.3. Quantitative Lab Markers

These biometric data points were collected at different time points to determine whether they are statistically significant. For the first lactate level after ECMO, the average was 5.70 mmol/L. The average first pH after ECMO was 7.27. Then, the same three markers were collected and measured 24 h post-CA. The average lactate level 24 h after cardiac arrest was 1.50 mmol/L. The mean pH 24 h after arrest was 7.37. Lastly, the average troponin level 24 h after arrest was 933,317.25 ng/L.

### 3.4. ECMO Outcomes

This data set details the outcomes of ECMO in 15 patients. Of the patients with gathered information, 10 survived until ECMO decannulation, and 5 did not. Eight patients survived to ICU and hospital discharge, seven did not. Of those eight, all survived for 30 days and 3 months after ECMO initiation. There was cannulation failure in 6 of 15 patients, for a success rate of 9. The mean ICU days was 9. As for ECMO complications (those arising directly from ECMO), results can be seen in Table 3.

Not included in the data tables, but categories in which data were also collected, were complications listed in the patient’s diagnosis, cannulation site, ECMO team composition, CPC at discharge, patient diagnosis (the primary diagnoses listed in the patient’s chart), and bleeding using the BARC scale. Multiple categories, such as CHF class (preserved or reduced ejection fraction) and infections after ECMO, had additional information but were not included in the statistical analysis.

The quantitative data with *p* < 0.05 were first lactate levels after ECMO cannulation and troponin levels 24 h after cardiac arrest. The qualitative data with *p* < 0.05 were CHF, survival to ECMO decannulation, survival to ICU discharge, and cannulation failure. Both 30-day ECMO survival and 3-month survival have statistically significant *p*-values but are not included in the list since they are after hospital survival, which is our dividing group. Survival to hospital discharge in the study meant that the patients removed from ECMO no longer needed hospital-level care and had a condition stable enough that ECMO and the hospital were no longer required. In addition, after three months of hospital discharge, survivors were required to attend an outpatient consultation with a specialist to help identify functional neurological recovery. Survival as a variable in this case does not consider the neurological status at discharge, neuron-specific enolase, or the need for at-home rehabilitation.

Analysis of parameters and data points from our table that were not included in our statistical analysis was also noted. For example, we were able to visualize the types of infections our patients acquired after ECMO. For instance, three patients developed Acinetobacter baumannii infections, one developed a wound infection from ECMO, and five others developed generalized infections such as influenza or pneumonia during ECMO. Although also not included in our data overview, the main cannulation sites were the arterial and femoral veins and arteries (both left and right). Regarding team composition, all 15 cases had an anesthesiologist/ECMO specialist; 15 also had a perfusionist; cardiothoracic surgeons were present in 8 implantations, cardiologists in 7, and nurses in 6. Lastly, information was also obtained regarding limb ischemia and vascular repair: one vascular reconstruction, one leg ischemia, one heel gangrene, and one femoral artery and vein reconstruction.

## 4. Discussion

This study is the first of its kind in Latvia, analyzing ECMO center operational metrics and identifying areas for improvement for our patients. By collecting data on many factors related to IHCA cases and their relationship with ECMO, we can obtain a clearer picture of challenges and implementation factors. This includes factors that did not go according to plan (such as bleeding or infection). First, earlier program activation would benefit our patients by reducing flow times after cardiac arrest. Secondly, a specialized team that is more selective in which patients receive ECMO based on rehabilitation potential would benefit hospital resource allocation. In other research, the SAVE score was developed to provide clinicians with a tool to predict survival for those on VA-ECMO in refractory cardiogenic shock [13]. Such a tool, when implemented in our hospital, could support ECMO patient selection and rationalize decision-making during IHCA cases. Lastly, streamlining the standardization of multidisciplinary decision-making would greatly benefit our patients by allowing multiple specialists to reach a clear, common decision.

In Latvia, the State Agency of Medicine published information about ECMO in January 2022. They state that the use of the technology in the medical field grew in popularity after the rise in SARS-CoV-2 in 2020. Due to many patients not receiving sufficient support from mechanical ventilation alone, ECMO was an imperative technology at the time [11]. With further development of the ECMO program at PSKUS, the technology is becoming increasingly available not only to cardiac surgery patients but also to others, such as those after large MVAs or those with unstable lung function. Currently, in Latvia, the development of portable ECMO systems for patients from district hospitals that lack the advanced technologies found in city-center clinical university hospitals is underway. This new service is in trial at Riga East Clinical University Hospital, in accordance with the state ambulance service [14].

A similar study was published in Spain, which analyzed an ECMO program for IHCA and OHCA patients. Their main results and conclusions were that, of their 54 patients, 16 (29.6%) were alive 180 days after the start of ECMO, and 15 (23.4%) had a favorable neurological status. In addition, ten patients who did not survive had their organs donated. The study found that the most common cause of cardiac arrest was due to acute coronary syndrome, then pulmonary embolism, followed by accidental hypothermia [15].

Other important topics in current research are rhythm types during cardiac arrest and the role of adrenaline administration during cardiac arrest. In a Romanian study, it was concluded that the lower the dose of adrenaline administered (up to 4 mg), the better the survival outcomes, compared with cases where more than 4 mg was given [16]. In our study, the average epinephrine dose was 4 mg. Regarding fatal cardiac rhythms, studies show that patient survival is lower when cardiac rhythm changes are present (26.5% survival) compared to (78.5% survival) when they are absent [17]. For comparison, our results showed that 37.5% of the survivor group had a mixed cardiac rhythm, whereas none in the non-survivor group did.

When looking at the data and comparing survivors and non-survivors, some differences were significant, and others were not. The first lactate level after ECMO was statistically significant (*p* = 0.035). One study found that pre-ECMO and peak lactate values were not statistically significant predictors of 30-day mortality. However, lactate and lactate clearance values at 24 h were prognostic for the 30-day survival outcome [18]. Lactate levels after CA rise due to an imbalance between oxygen supply and demand, thus there is an inequality in production and balance of the substance [19]. Another published study indicated that lactate levels were consistently measured higher in the non-survivor group (12 h lactate > 8.2 mmol/L and 24 h lactate > 2.6 mmol/L) than in the survivor group [20]. Additionally, ECMO flow time delays >30 min and lactate levels >8.0 mmol/L were strong predictors of patient mortality [21]. Similarly, lactate levels 24 h after cardiac arrest were significant in our study. However, given the small sample size and lack of neurological outcome assessment, they warrant further study in our population. Another quantitative data category, significant at *p* = 0.005, were troponin levels 24 h after arrest. A similar finding in an article stated that a peak troponin level of 400 ng/mL correctly identified patients who would be weaned from venoarterial ECMO roughly 90% of the time [22]. However, another published study found no statistical significance in troponin T levels or in the 90-day survival rate after venoarterial ECMO implantation [23]. Given the small sample size, we recommend revisiting the existing data to assess reproducibility, as the study included only 102 patients.

In terms of other numerical data, there was no statistical significance for the following categories: age, BMI, epinephrine dose during resuscitation measures, collapse to ECMO (low-flow) time, first lactate and pH post-CA, lactate, and pH 24 h after CA, first pH after ECMO, first troponin levels 24 h post-arrest, and ICU days. Even though these data sets were not statistically significant, they still provided valuable information for us and our program. For instance, the transition to ECMO gave us a good insight into how long it takes to place ECMO after receiving the call that it is needed. Our goal is to have ECMO placed within 60 min of call time, although this is not always possible. As shown, the maximum time was 75 min, the average was 44 min, and Q3 was 50 min. This is still acceptable as our response time, since it is subject to factors beyond our control, such as traffic on the road, until an ECMO specialist arrives at the hospital from their residence after being on-call.

Furthermore, the qualitative data were informative and provided insights into possible predictors of patients who survived to hospital discharge. Of the patient history data, the only significant finding was CHF (*p*-value = 0.032). This can be interpreted so that patients with CHF were more likely to survive than those without CHF (87.5% vs. 20%). Cautious interpretation of this finding is warranted, given the lack of explanation in the literature and the small patient group of the study. Continuing forward, significant *p*-value-based data (in correlation with survival to hospital discharge) included survival to ECMO decannulation (*p* = 0.007), survival to ICU discharge (*p* < 0.001), and cannulation failure (*p* = 0.041). Cannulation failure is the inability to establish and sustain extracorporeal circulation despite attempts to gain vascular access, due to anatomical, technical, or procedural limitations, prior to the initiation of ECMO flow. For ECMO decannulation, 71.4% of non-survivors did not reach ECMO removal. A total of 100% of survivors of hospital discharge survived ICU discharge, and vice versa, meaning no patient in the study group passed away after ICU discharge while still in the hospital. In terms of cannulation failure, 28.6% of those who did not survive had cannulation failure, while only 12.5% of survivors had cannulation failure. Two other statistically significant data sets were 3-month ECMO survival (*p* value < 0.001) and 3-month survival (*p* value < 0.001). However, these points, compared with survival from hospital discharge, do not have meaningful clinical value, since most patients in our study were not on ECMO long enough to reach 30 days or 3 months; thus, all who survived 1 month or 3 months were already discharged from the hospital. All survivors lived to 30 days, and all non-survivors did not; the same applied to 3-month survival. All other qualitative data were not significant in this case.

Our study had both strengths and limitations. Firstly, the sample size of our study was relatively small, with only fifteen participants. This was due to a criterion that included only IHCA and was based on a year of ECMO patients, which is not comparable to larger cities, since Riga’s population is smaller. No more patients met our criteria. Another limitation was the study design: it was retrospective and observational, with missing data at times and documentation that was not always clear. It would also have been beneficial to have a control group, but that was not possible in this case. As for strengths, we are proud to be the first in Latvia to report our ECMO research regarding the first-year outcomes of a newly updated program. The design process also ensured that all patients had signed informed consent forms prior to data collection and analysis. Overall, this study gave an insight into our hospital’s ECMO/ECPR program and clinical characteristics that were observed.

## 5. Conclusions

This study highlights the first structured year of the ECMO program at Pauls Stradins Clinical University Hospital. Findings confirm that even in smaller countries (where resource constraints may be greater than in other countries), ECMO centers can be established and well-maintained. This study allowed us to look back on a year of cases, analyze them, and identify opportunities for future improvement to better our team, hospital, and community for our ECMO patients. While the study of the design yielded new insights, it would have benefited from a larger sample and a more robust methodology. However, this is hard to achieve in a smaller country with relatively few ECMO patients. In the future, a study with a larger cohort and standardized neurological assessments would be beneficial to evaluate clinical outcomes in an unbiased manner. In addition, we would like further to analyze ECMO fatalities in conjunction with organ donation opportunities. As the first-ever study of an ECMO program in Latvia, this study contributes data to the limited scientific literature on such programs in smaller countries. We hope this study sheds light on the performance of our ECMO center, providing insight to other departments, hospitals, and countries, and helping them further develop their own ECMO programs.

## Figures and Tables

**Table 1:** Quantitative Variables and Survival to Hospital Discharge.

Variable	Total (*n* = 15) Median (IQR)	Survivors (*n* = 8) Median (IQR)	Non-Survivors (*n* = 7) Median (IQR)	*p*-Value
Age (years)	65.00 (61.00–72.00)	66.50 (63.00–69.50)	65.00 (48.00–79.00)	0.694
BMI (kg/m^2^)	27.00 (26.70–30.90)	27.80 (26.80–36.90)	27.00 (22.70–30.90)	0.303
Epinephrine Dose (mg)	6.00 (4.00–7.00)	5.00 (2.00–8.00)	6.00 (1.00–7.00)	0.138
Collapse to ECMO (min)	45.00 (40.00–50.00)	44.00 (40.00–47.50)	45.00 (30.00–75.00)	0.463
First Lactate Post-Arrest (mmol/L)	12.80 (8.40–16.30)	9.45 (8.45–17.00)	12.90 (6.40–15.50)	0.065
First Lactate After ECMO (mmol/L)	7.03 (7.00–7.17)	7.09 (7.00–7.23)	7.03 (6.60–7.28)	**0.035**
First pH Post-Arrest	12.40 (9.20–13.70)	11.40 (9.00–13.00)	12.40 (8.60–17.00)	0.836
pH 24 Hours Post-Arrest	7.35 (7.31–7.37)	7.37 (7.32–7.42)	7.35 (7.20–7.44)	0.366
Lactate 24 Hours Post-Arrest (mmol/L)	3.20 (1.10–8.70)	0.80 (1.00–1.30)	3.20 (0.45–2.10)	0.181
First pH After ECMO	7.13 (7.04–7.33)	7.18 (7.06–7.34)	7.13 (7.00–7.36)	0.205
First Troponin After Arrest (ng/L)	6554.94 (901.36–26204.00)	3386.47 (115.42–73210.25)	8654.94 (287.17–15445.39)	0.813
Troponin 24 Hours Post-Arrest (ng/L)	465830.00 (312068.23–941600.00)	348301.00 (479.46–953172.23)	933317.25 (221.27–1111801.1)	**0.005**
ICU Days	2.00 (2.00–25.00)	7.00 (2.00–14.00)	2.00 (2.00–23.00)	0.232

Bold *p*-values indicate statistical significance (*p* < 0.05). *p*-values calculated using Mann-Whitney U test.

**Table 2:** Patient Comorbidities and Survival Outcomes.

Comorbidity	Total (*n* = 15) *n* (%)	Survivors (*n* = 8) *n* (%)	Non-Survivors (*n* = 7) *n* (%)	*p*-Value
Diabetes Mellitus	7 (46.7%)	3 (37.5%)	4 (57.1%)	0.608
COPD	2 (13.3%)	1 (12.5%)	1 (14.3%)	>0.99
Malignancy	2 (13.3%)	1 (12.5%)	1 (14.3%)	>0.99
Previous MI	3 (20.0%)	2 (25.0%)	1 (14.3%)	>0.99
Dyslipidemia	11 (73.3%)	6 (75.0%)	5 (71.4%)	>0.99
Previous Stroke	1 (6.7%)	1 (12.5%)	0 (0.0%)	>0.99
Chronic Kidney Disease	4 (26.7%)	3 (37.5%)	1 (14.3%)	0.569
Chronic Heart Failure	8 (53.3%)	7 (87.5%)	1 (14.3%)	**0.032**
Hypertension	9 (60.0%)	6 (75.0%)	3 (42.9%)	0.315
Current Smoker	2 (13.3%)	2 (25.0%)	0 (0.0%)	0.467

Bold values indicate statistical significance (*p* < 0.05). *p*-values calculated using Fisher’s exact test.

**Table 3:** Qualitative Variables and Survival to Hospital Discharge.

Variable	Category	Total (*n*=15) *n* (%)	Survivors (*n* = 8) *n* (%)	Non-Survivors (*n* = 7) *n* (%)	*p*-Value
Gender	Male	12 (80.0%)	8 (100.0%)	4 (57.1%)	0.077
	Female	3 (20.0%)	0 (0.0%)	3 (42.9%)	
Diagnosis	Acute PCA	8 (53.3%)	4 (50.0%)	4 (57.1%)	0.056
	Elective PCA	4 (26.7%)	4 (50.0%)	0 (0.0%)	
	Post-cardiac surgery	3 (20.0%)	0 (0.0%)	3 (42.9%)	
ECMO Complications	Yes	9 (60.0%)	5 (62.5%)	4 (57.1%)	>0.99
Initial Rhythm	SR	14 (93.3%)	8 (100.0%)	6 (85.7%)	0.467
	AF	1 (6.7%)	0 (0.0%)	1 (14.3%)	
Rhythm Before ECPR	PEA	6 (40.0%)	3 (37.5%)	3 (42.9%)	0.408
	Mixed	3 (20.0%)	3 (37.5%)	0 (0.0%)	
	Ventricular	2 (13.3%)	1 (12.5%)	1 (14.3%)	
	VF	2 (13.3%)	1 (12.5%)	1 (14.3%)	
	VT	1 (6.7%)	0 (0.0%)	1 (14.3%)	
	Asystole	1 (6.7%)	0 (0.0%)	1 (14.3%)	
Survival to ECMO Decannulation	Yes	10 (66.7%)	8 (100.0%)	2 (28.6%)	**0.007**
Survival to ICU Discharge	Yes	8 (53.3%)	8 (100.0%)	0 (0.0%)	**<0.001**
30-Day Survival	Yes	8 (53.3%)	8 (100.0%)	0 (0.0%)	**<0.001**
3-Month Survival	Yes	8 (53.3%)	8 (100.0%)	0 (0.0%)	**<0.001**
Cannulation Failure	Yes	6 (40.0%)	1 (12.5%)	5 (71.4%)	**0.041**
Stroke	Yes	2 (13.3%)	1 (12.5%)	1 (14.3%)	>0.99
Limb Ischemia/Vascular Repair	Yes	4 (26.7%)	2 (25.0%)	2 (28.6%)	>0.99
Infection	Yes	7 (46.7%)	4 (50.0%)	3 (42.9%)	>0.99
AKI/CRRT	Yes	1 (6.7%)	1 (12.5%)	0 (0.0%)	>0.99

Bold *p*-values indicate statistical significance (*p* < 0.05). *p*-values calculated using Fisher’s exact test.

## Data Availability

The raw data supporting the conclusions of this article will be made available by the authors on request.

## Abbreviations

The following abbreviations are used in this manuscript:

ACS	Acute coronary syndrome
AF	Atrial fibrillation
AKI	Acute kidney injury
BARC	Bleeding Academic Research Consortium
CA	Cardiac arrest
CPC	Cerebral performance score
CPR	Cardiopulmonary resuscitation
CHF	Chronic heart failure
CI	Confidence interval
CRRT	Continuous renal replacement therapy
CRT-D	Cardiac resynchronization therapy defibrillator
ECG	Electrocardiogram
ECPR	Extracorporeal cardiopulmonary resuscitation
ECMO	Extracorporeal membrane oxygenation
ER	Emergency room
EtCO_2_	End-tidal carbon dioxide
ICD	Implantable cardioverter defibrillator
ICU	Intensive care unit
IHCA	In-hospital cardiac arrest
MVA	Motor vehicle accident
OHCA	Out-of-hospital cardiac arrest
PCA	Percutaneous coronary angioplasty
PEA	Pulseless electrical activity
PSKUS	Pauls Stradins Clinical University Hospital
ROSC	Return of spontaneous circulation
SARS-CoV-2	Severe acute respiratory syndrome coronavirus 2
SR	Sinus rhythm
VA	Venoarterial
VF	Ventricular fibrillation
VT	Ventricular tachycardia
VV	Venovenous
